# Dopaminergic Dysfunction in Mammalian Dopamine Neurons Induced by Simazine Neurotoxicity

**DOI:** 10.3390/ijms18112404

**Published:** 2017-11-13

**Authors:** Xueting Li, Jia Yu, Jianan Li, Yanping Wu, Baixiang Li

**Affiliations:** 1 Department of Toxicology, College of Public Health, Harbin Medical University, Harbin 150081, China; lxt0451@163.com (X.L.); lijianan0468@163.com (J.L.); wuyanpinghrb@163.com (Y.W.); 2 Department of Environmental Health, College of Public Health, Harbin Medical University, Harbin 150081, China; yujia991@163.com

**Keywords:** MN9D cells, herbicide simazine, dopamine, metabolism, neurodegenerative disease

## Abstract

Many studies have shown that the pollutant simazine (6-chloro-*N*,*N*′-diethyl-1,3,5-triazine-2,4-diamine), which has been overused, inhibits the proliferation of mammalian dopaminergic cells, and affects the developmental differentiation of mammalian dopaminergic neurons. However, few studies have shown the effects of simazine on dopaminergic metabolism in these cells. Therefore, we aim to examine the metabolic effects of simazine exposure in mouse dopaminergic progenitor neurons (MN9D) at different exposure times. The cells were treated with simazine at 0, 150, 300 and 600 µM for 12, 24 and 48 h, respectively. The content of dopamine in these cells was then examined using the enzyme-linked immunosorbent assay (ELISA) kit. Real-time quantitative polymerase chain reaction (PCR) and western blotting were performed to analyze the mRNA and protein expression of aromatic amino acid decarboxylase (AADC), tyrosine hydroxylase (DYT5b), dopamine transporter (DAT), monoamine vesicular transporter 2 (VMAT2), monoamine oxidase (MAO) and catechol-*O*-methyl transferase (COMT). The results showed that simazine influenced the metabolism of dopamine and led to a decrease in dopamine level in these cells which may eventually lead to neurological disorders of the dopaminergic system.

## 1. Introduction

Dopamine is an important neurotransmitter in the mammalian brain and participates in the regulation of emotional, cognitive, memory, and other physiological functions of the central nervous system [1]. Numerous animal studies have concentrated primarily on the effects of simazine on the reproductive and endocrine systems [2,3]. Extensive application of herbicides has not only caused environmental pollution, but has also endangered human health. One of the most dangerous water pollutants is triazine herbicides, used worldwide as residual nonselective herbicides to control broad-leaved weeds and annual grasses [4]. Due to its high potency and broad spectrum of activity, the use of atrizine was gradually replaced by simazine in the 1960s. Simazine is used to extirpate weeds in agriculture and is one of the triazine herbicides. In 2002, the amount of simazine applied in the US alone was more than 2000 tons. The US Environmental Protection Agency set the limit value of simazine in drinking water at <4 µg/L, Japan set it at <3 µg/L and the European safety level in surface water is 0.1 mg/L [5]. However, simazine was detected at a concentration of approximately 13 µg/L in Chinese river samples [6]. The huge amounts applied and the diverse exposure routes of simazine have increased the risk to human health. Moreover, simazine is considered to be slightly soluble and nontoxic, is difficult to degrade in the natural environment, and has multiple exposure pathways, including water, soil, and the food chain [7,8,9,10]. Simazine has already been detected at high concentrations in rainwater, surface water and underground water in France, the US and Spain, and has even been detected in food such as tomatoes and living fish [11,12,13]. Zhou L. et al. found that the amount of simazine in fruit and vegetable samples such as apples and pears ranged from 10.8 to 25.2 ng/mL. Simazine was significantly associated with wheeze in male farmers from North Carolina and Iowa [14]. At present, studies mainly focus on the mutagenicity, reproductive developmental toxicity and immunotoxicity of simazine [15,16,17,18]. Researchers found that lengthy exposure to low doses of simazine can influence the development of early life stages in mammals, where it can act as a neural endocrine disruptor and increase immunotoxicity [19,20]. In addition, some studies found that simazine affected dopamine synthesis via an alteration in the synthetic enzyme, tyrosine hydroxylase (DYT5b), in rattus norvegicus adrenal pheochromocytoma (PC12) cells and destroyed the homeostasis of the dopamine system [21]. However, few studies have investigated the neurotoxicity of simazine on the metabolism of mammalian dopaminergic neurons. Previous research by our group found that simazine influenced development-related gene expression in mouse dopaminergic progenitor neurons (MN9D). In addition, exposure to 300 and 600 µM simazine for 24 and 48 h resulted in the up-regulation of development-related gene expression and dopaminergic damage in these cells, which is related to neurodegenerative disease [22].

Dopaminergic neurons are crucial in adjusting motor behavior, working memory and other physiological processes. They mainly assemble in the substantial nigra zona compacta (SNc) and ventral tegmental area (VTA) [23]. The dysfunction of dopaminergic neurons may result in neurodegenerative diseases. Dopaminergic neurons in the SNc primarily function by the nigrostriatal pathway, while those in the VTA function by the mesolimbic pathway and mesocortical pathway. These pathways are controlled by genes, such as tyrosine hydroxylase and dopamine transporter (DAT) [24]. In the present study, the important genes involved in the differentiation and survival of MN9D cells, and the synthesis, secretion, and reuptake of dopamine were selected to determine the effects of simazine on their metabolism which can lead to dopaminergic damage in these neurons.

## 2. Results

### 2.1. Effects of Simazine on Mouse Dopaminergic Progenitor Neurons (MN9D) Viability

**Figure 1:**
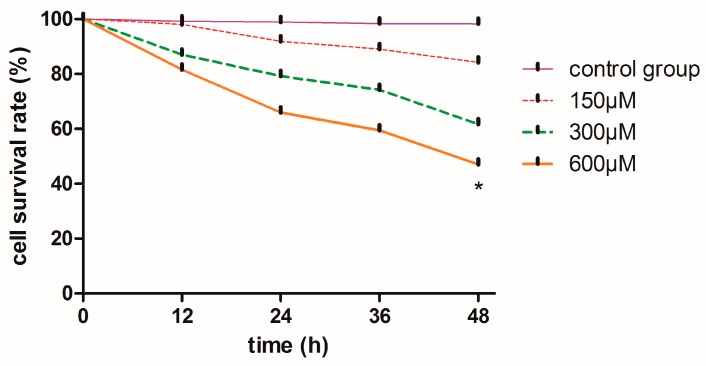
Effects of simazine on mouse dopaminergic progenitor neurons (MN9D) viability was assessed by Cell Counting Kit (CCK)-8 assay. Data represent absorbance values as percentages of untreated control cells, * statistically significant difference compared with the control, * *p* < 0.05, 3 repeated experiments for each group, *n* = 3.

The viability of MN9D cells after treatment with 600 µM simazine for 48 h decreased to 50%, which was significantly reduced compared with the control (0.5% *w*/*v* phosphate buffer solution, PBS) (*p* < 0.05) (Figure 1).

### 2.2. Effects of Simazine on mRNA Levels in MN9D Cells

**Figure 2:**
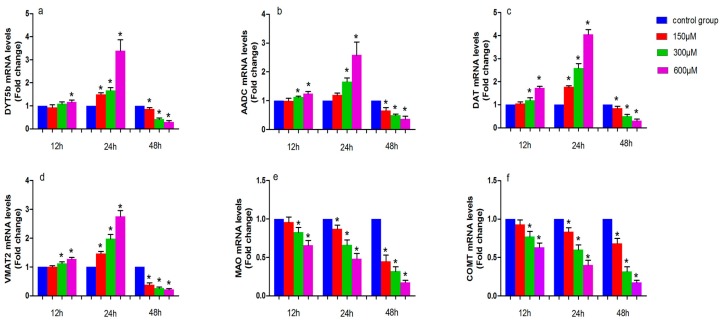
The effects of exposure to simazine for 12, 24, and 48 h on tyrosine hydroxylase (DYT5b) (**a**); aromatic amino acid decarboxylase (AADC) (**b**); dopamine transporter (DAT) (**c**) and monoamine vesicular transporter 2 (VMAT2) (**d**); monoamine oxidase (MAO) (**e**); catechol-*O*-methyl transferase (COMT) (**f**) mRNA relative levels fold change to control in MN9D cells were presented. Bars indicate mean ± S.E.M. * statistically significant difference compared with the control, * *p* < 0.05, 3 repeated experiments for each group, *n* = 3.

The levels of tyrosine hydroxylase (DYT5b), aromatic amino acid decarboxylase (AADC), dopamine transporter (DAT), monoamine vesicular transporter 2 (VMAT2), monoamine oxidase (MAO) and catechol-*O*-methyl transferase (COMT) mRNA in simazine-treated MN9D cells were determined. We analyzed the main effects of exposure dose, exposure time and the interaction of these two factors. All gene mRNA levels were regulated in a time- and dose-dependent manner (Figure 2).

Figure 2 shows the effect of simazine exposure for 12 and 24 h on *DYT5b* (a), *AADC* (b), *DAT* (c) and *VMAT2* (d), *MAO* (e), *COMT* (f) mRNA levels. *DYT5b* (a) mRNA levels was significantly increased following exposure to 600 µM simazine for 12 h compared with the control (*p* < 0.05); all dose groups of *DYT5b* mRNA levels for 24 h were significantly increased compared with the control (*p* < 0.05); *AADC* (b) mRNA level were significantly increased following exposure to 300 and 600 µM simazine for 12 and 24 h compared with the controls (*p* < 0.05). *DAT* (c) and *VMAT2* (d) mRNA levels were significantly increased following exposure to 300 and 600 µM simazine for 12 h compared with the controls (*p* < 0.05); *DAT* and *VMAT2* mRNA levels were also significantly increased following exposure to 150, 300 and 600 µM simazine for 24 h compared with the controls (*p* < 0.05) (c and d). *MAO* and *COMT* mRNA levels in cells exposed to 300 and 600 µM simazine for 12 h were significantly decreased compared with the controls (*p* < 0.05) and exposed to the same dose of simazine for 24 h were also significantly decreased compared with the controls (*p* < 0.05) (d and e). However, the mRNA levels of these genes were significantly decreased following exposure to simazine for 48 h compared with the controls (*p* < 0.05).

### 2.3. Effects of Simazine on Protein Expression in MN9D Cells

**Figure 3:**
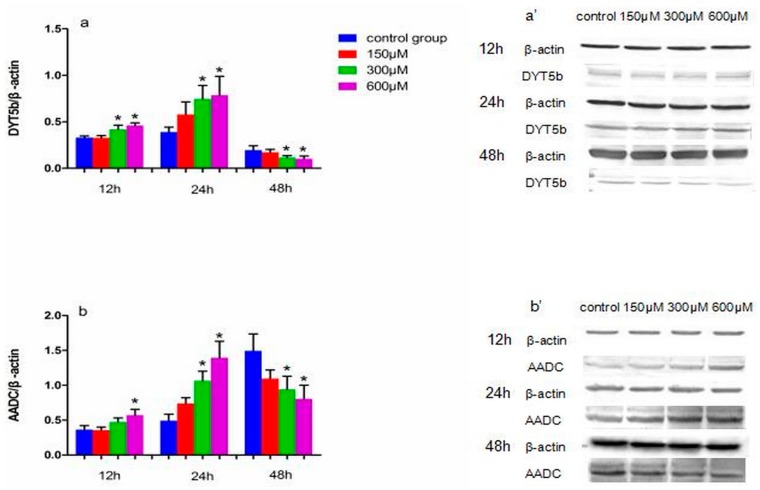
The effects of exposure to simazine for 12, 24, 48 h on the expression of DYT5b (**a**,**a′**), AADC (**b**,**b′**) protein in MN9D cells determined with a western blotting analysis. Bars indicate mean ± S.E.M. * statistically significant difference compared with the control, * *p* < 0.05, 3 repeated experiments for each group, *n* = 3.

**Figure 4:**
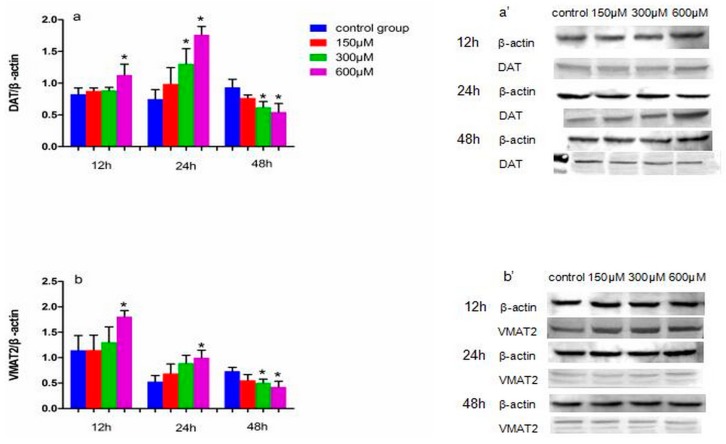
The effects of exposure to simazine for 12, 24, 48 h on expression of DAT (**a**,**a′**), VMAT2 (**b**,**b′**) protein in MN9D cells determined with a western blotting analysis. Bars indicate mean ± S.E.M. * statistically significant difference compared with the control, * *p* < 0.05, 3 repeated experiments for each group, *n* = 3.

**Figure 5:**
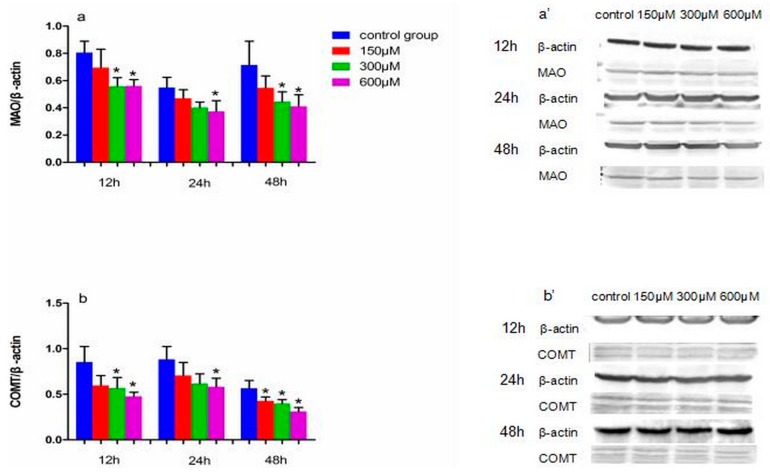
The effects of exposure to simazine for 12, 24, 48 h on expression of MAO (**a**,**a′**), COMT (**b**,**b′**) protein in MN9D cells determined with a western blotting analysis. Bars indicate mean ± S.E.M. * statistically significant difference compared with the control, * *p* < 0.05, 3 repeated experiments for each group, *n* = 3.

The levels of DYT5b, AADC, DAT, VMAT2, MAO and COMT protein expression in simazine-treated MN9D cells were determined. We analyzed the main effects of exposure dose, exposure time and the interaction of these two factors (Figure 3, Figure 4 and Figure 5).

Figure 3 shows the effect of exposure to simazine for 12 and 24 h on the protein expression levels of DYT5b (a and a′) and AADC (b and b′). DYT5b protein levels in cells exposed to 300 and 600 µM simazine for 12 h were significantly increased compared with the control (*p* < 0.05) (a and a′); AADC protein levels in cells exposure to 600 µM simazine for 12 h was significantly increased compared with the control (*p* < 0.05) (b and b′); DYT5b and AADC protein levels in cells exposed to 300 and 600 µM simazine for 24 h were significantly increased compared with the controls (*p* < 0.05). DYT5b, AADC protein levels following exposure to 300 and 600 µM simazine for 48 h were all significantly decreased compared with the controls (*p* < 0.05).

Figure 4 shows the effect of exposure to simazine for 12 and 24 h on the protein expression levels of DAT (a and a′) and VMAT2 (b and b′). DAT and VMAT2 protein levels in cells exposed to 600 µM simazine for 12 h were significantly increased compared with the controls (*p* < 0.05); DAT protein levels in cells exposed to 300 and 600 µM simazine for 24 h were significantly increased compared with the control (*p* < 0.05) (a and a′); VMAT2 protein levels in cells exposed to 600 µM simazine for 24 h were significantly increased compared with the control (b and b′); the protein levels of the two following genes exposed to 300 and 600 µM simazine for 48 h were significantly decreased compared with the controls (*p* < 0.05).

The protein expression of MAO and COMT following exposure to simazine is shown in Figure 5. MAO (a and a′) and COMT (b and b′) protein levels following exposure to 300 and 600 µM simazine for 12 h and following exposure to 600 µM simazine for 24 h were significantly decreased compared with the controls, respectively (*p* < 0.05); MAO protein levels following exposure to 300 and 600 µM simazine for 48 h and COMT protein levels following exposure to all doses groups for 48 h were significantly decreased compared with the controls, respectively (*p* < 0.05).

### 2.4. Immunofluorescence Detection of DYT5b and DAT in Simazine-Treated MN9D Cells

**Figure 6:**
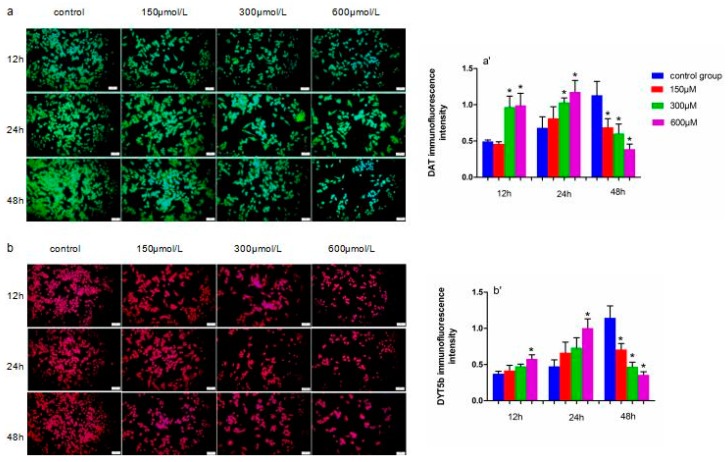
Representative images of DAT (green) (**a**,**a′**), DYT5b (red) (**b**,**b′**) protein in MN9D cells exposed to simazine for 12, 24 and 48 h detected with immunofluorescence were presented, together with inmmunofluorescence intensity. Bars indicate mean ± S.E.M. * statistically significant difference compared with the control, * *p* < 0.05, 3 repeated experiments for each group, *n* = 3. Scale bars, 25 µm.

Immunofluorescence staining was used to detect DYT5b and DAT protein expression, which are markers of dopaminergic neurons (Figure 6). We analyzed the mean fluorescence intensity to determine DYT5b and DAT protein expression levels. DAT protein levels in cells exposed to 300 and 600 µM simazine for 12 and 24 h were significantly increased compared with the controls (*p* < 0.05) (a and a′); DYT5b protein levels in cells exposed to 600 µM simazine for 12 and 24 h were significantly increased compared with the controls (*p* < 0.05) (b and b′). DYT5b and DAT protein levels in cells exposed to all doses of simazine for 48 h were significantly decreased compared with the controls (*p* < 0.05) (Figure 6).

### 2.5. Dopamine Levels in Simazine-Treated MN9D Cells

**Figure 7:**
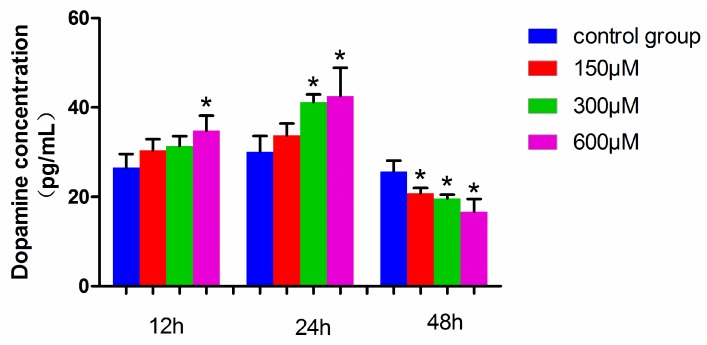
Dopamine levels in MN9D cells exposed to simazine for 12, 24, 48 h. Bars indicate mean ± S.E.M. * statistically significant difference compared with the control, * *p* < 0.05, 3 repeated experiments for each group, *n* = 3.

The dopamine level in MN9D cells exposed to 600 µM simazine for 12 h was significantly increased compared with the control (*p* < 0.05), and those exposed to 300 and 600 µM simazine for 24 h were significantly increased compared with the control (*p* < 0.05). However, the dopamine levels of all the dose groups for 48 h simazine exposure were significantly decreased compared with the controls (*p* < 0.05) (Figure 7). These results showed that the dopamine levels in MN9D cells were affected by the interactions of exposure time and dose (*p* < 0.05).

## 3. Discussion

Simazine can be detected in soil and ground water samples due to its overuse and the toxicity of simazine urgently requires further in-depth studies. We attempted to assess the effects of simazine on the synthesis and metabolism of dopaminergic neurons. Dopamine synthesis and transfer disorders may lead to the onset of Parkinson’s disease (PD), Alzheimer’s disease (AD) and other common neurological disorders [25,26,27]. The neurotoxicity of simazine on the dopaminergic system is unclear. There are very few in vitro studies on the dopaminergic system. The MN9D cell line is a mouse dopaminergic neuron line, and was selected for this study to determine the influence of simazine on dopaminergic neuron synthesis and metabolism. The metabolism of dopamine includes its synthesis, storage, release, reuptake and inactivation. Tyrosine in catecholamine neurons is converted to *l*-DOPA following the catalysis of DYT5b. Then *l*-DOPA is converted to dopamine by AADC. After the synthesis of dopamine in dopaminergic neurons, dopamine is transported and stored in vesicles by VMAT2 [28]. When the action potential reaches the nerve endings, dopamine is released in the synaptic cleft and functions by binding with the postsynaptic receptors. DAT can reuptake dopamine from the synaptic cleft into presynaptic membranes and then dopamine is stored in the vesicles by VMAT2. DA in the synaptic cleft is transformed into the final product by the enzymolysis of COMT and MAO. Under normal circumstances, each factor plays a role in maintaining a steady state in the dopamine system. When any of these factors are affected, this can disrupt the balance in the dopaminergic system and cause a series of physiological effects [29,30]. DYT5b is a key rate-limiting enzyme. Positive neurons are able to produce either *l*-DOPA or dopamine in target areas of ventral midbrain dopaminergic neurons. Keber suggested that striatal tyrosine hydroxylase^+^ cells may synthesize dopamine cooperatively and subsequently contribute to supraphysiological concentrations of synaptic dopamine [31]. In our study, we observed that the mRNA and protein expression of DYT5b in cells exposed to simazine for 12, 24, and 48 h was significantly up-regulated after 12 and 24 h exposure and significantly down-regulated after 48 h exposure (Figure 2). DYT5b had an immediate effect on the synthesis of *l*-DOPA. As a result, the content of dopamine changed. AADC is a homodimeric pyridoxal phosphate-dependent enzyme responsible for the synthesis of dopamine. AADC, which converts *l*-DOPA to dopamine, is considered the rate-limiting enzyme for the synthesis of biogenic amine. Researchers found that monoamines synthesized by DYT5b-AADC may compensate for lost neurotransmitters following spinal cord injury and may play specific roles in the recovery of sensory, motor and autonomic functions. In addition, some studies have shown that AADC deficiency is a rare pediatric neuro-metabolic disease and defects in AADC result in neurotransmitter deficiencies. DYT5b and AADC are crucial in dopamine metabolism [32]. In our study, the mRNA and protein levels of AADC in MN9D cells were consistent with those of DYT5b.

DAT is synthesized and expressed by the soma, dendrite and axon of dopaminergic neurons and is distributed on the membrane of dendrites and axons. The main function of DAT is the reuptake of dopamine in the synaptic cleft. Due to its specificity for dopamine, the content of DAT can reflect dopaminergic system function. Therefore, many studies have evaluated the presynaptic function of dopaminergic neurons using DAT. Some studies have reported the complex regulation of DAT and its effects on the regulation of other proteins [24,33,34,35]. The main function of VMAT2 is to store dopamine in the vesicles. The inhibition of VMAT2 contributes to dopaminergic neuron death and recent evidence has suggested that the vesicular storage of dopamine may contribute to the death of nigral neurons in PD. Therefore, VMAT2 plays a leading role in storing dopamine in vesicles to avoid degradation by MAO. Approximately 80% of monoamine neurotransmitters are reabsorbed by nerve terminals and reuptake is the main way to suspend the physiological effects of monoamine neurotransmitters; thus, DAT and VMAT2 are crucial [36,37,38]. In the present study, we found that following exposure to simazine for 12 and 24 h, DAT and VMAT2 expression increased in MN9D cells and after 48 h exposure, DAT and VMAT2 expression significantly decreased (Figure 2 and Figure 4). The variation in expression of DAT and VMAT2 changed the transport and storage capacity and inhibited the reuptake of dopamine into vesicles. This inhibition could increase the accumulation of dopamine in the cytoplasm and induce oxidative stress and toxicity leading to the death of dopaminergic neurons. Dopamine levels in MN9D cells were observed to follow the trends in mRNA and protein expression of DYT5b, AADC, DAT and VMAT2. Thus, we speculate that simazine affected the metabolism of dopamine in dopaminergic neurons by influencing DYT5b, AADC, DAT and VMAT2 mRNA and protein expression to change the content and activity of dopamine.

COMT and MAO are important degrading enzymes of dopamine. Recently, COMT has attracted strong neuroscientific interest due to its implication in dopaminergic neurotransmission [39]. Some studies have observed that these two genes were associated with cognitive function [40,41]. MAO and COMT inhibitors are the current optimal form of PD treatment and for maintaining monoamine balance [42]. The effects of COMT and MAO on dopamine are different due to their location. In our study, we observed that the mRNA and protein expression of COMT and MAO showed a significant downtrend after exposure to simazine for 12, 24, and 48 h. Therefore, the increase in AADC may have led to a rise in production, while decreasing levels of COMT and MAO decelerated the degradation of dopamine in MN9D cells. In this study, MAO and COMT showed a downward trend in these cells following exposure to simazine for 12 and 24 h, but dopamine levels in the cells showed an upward trend following exposure to simazine for 12 and 24 h. MAO and COMT had no significant effect on dopamine levels in the cells. Therefore, we speculate that simazine affects the synthesis and metabolism of dopamine by influencing DYT5b, AADC, DAT and VMAT2 expression in the MN9D dopaminergic neurons. Simazine is neurotoxic and acts on the mammalian dopaminergic metabolism in dopaminergic neurons. It can influence the synthesis, transport and metabolism of dopamine and leads to dysfunction in the natural balance of the dopaminergic system. Exposure time of 12 and 24 h and low-dose simazine exposure may increase dopamine levels in MN9D cells. However, the metabolism of dopamine may be damaged in MN9D cells after 48 h due to low-dose simazine exposure and dopamine levels were decreased. In general, an exposure time longer than 24 h to simazine, a harmful environmental pollutant, may cause a reduction in dopamine level and eventually lead to neurodegenerative diseases.

## 4. Materials and Methods

### 4.1. Materials

Simazine (98% pure) was obtained from Zhongshan Chemical Co., Ltd. (Zhejiang, China). Solutions of simazine used for cell treatment were prepared at various levels (150, 300 and 600 µM) by dissolving in 0.01 M, pH7.4 PBS (Solarbio, Beijing, China). The MN9D cell line was purchased from Shanghai Jining Biological Technology Ltd. (Shanghai, China). The cells were cultured in minimum essential medium (Hyclone, Logan, UT, USA), supplemented with 10% (*v*/*v*) heat-inactivated fetal calf serum (PAN Biotech, Aidenbach, Germany), and 100 units/mL penicillin-streptomycin in a cell culture incubator with 5% CO_2_ at 37 °C. The cells were cultured in 75 cm culture flasks and harvested when approximately 90% confluent. RNA and proteins were collected for further study.

### 4.2. Cell Viability Assay

Cell viability was measured using the Cell Counting Kit (CCK)-8 assay (CK04, Dojindo, Tokyo, Japan) to detect living cells. MN9D cells inoculated in 96-well plates were treated with 150, 300, or 600 µM simazine for 12, 24, or 48 h, respectively. Then CCK8 reagent (0.5 mg/mL) was added to the cell culture medium and incubated at 37 °C for 2 h. The content of reduced formazan is proportional to the number of living cells. The absorbance of formazan was evaluated at 450 nm using a microplate reader, Bio-Tek Elx800 (Bio-Tek, Winooski, VT, USA).

### 4.3. Total RNA Isolation and Quantitative Real-Time Polymerase Chain Reaction (PCR)

The total RNA separated from the cells was isolated using TRIzol^®^ reagent following the manufacturer’s instructions. The concentration of RNA was measured with an ND-2000C spectrophotometer (Thermo Scientific NanoDrop Products, Wilmington, DE, USA). PrimeScript^®^ RT with a gDNA eraser reagent kit (TaKaRa Biotechnology Co., Ltd., Tokyo, Japan) was used to synthetize cDNA with 1 µg of total RNA following the operating instructions. The PCR primers were designed and synthesized (TaKaRa Biotechnology Co., Ltd.) as the followed: β-actin: forward GGGAAATCGTGCGTGAC, reverse AGGCTGGAAAAGAGCCT; *DYT5b*: forward AAGCCTTCAGCTCCCCATTCT, revers CCCAGTTCTCCCAGGACATTG; *AADC*: forward CAGTCCTCCTCTTCACCC, revers CCACATCCTGCTGTTCTT; *MAO*: forward TAATGGACGGGAGATAAA, revers ATTGAAGATGAGGAGGCT; *COMT*: forward GCCGTCCACCACTTTCAT, revers ACCGCTACCTTCCAGACA; *DAT*: forward TCCACTAGCTGGCGGTCTTTC, revers GCCCCTGCTTCCTTCTGTATG; *VMAT2*: forward GCAGTCTGGATTTCCGTAGTATTTT, revers TCCTGTTCATCGTGTTCCTCG. The cDNA was amplified using the SYBR Green method (SYBR^®^ Premix Ex TaqTM II, TaKaRa Biotechnology Co., Ltd.) in an ABI 7500 Real-Time PCR system (Thermo Scientific, Hudson, NY, USA). The cycling conditions included denaturation at 95 °C for 5 s, 45 cycles of annealing at 55 °C for 34 s and then extension at 72 °C for 30 s. The cycle at which the sample fluorescence reached a threshold was defined as the threshold cycle (CT). The results were expressed as the relative expression ratio calculated on the basis of the real-time PCR efficiency and ΔCT. The ΔCT value for each gene (target or reference) was calculated by subtracting the CT value of the target sample from that of the control sample. The ratio of target gene expression in the treatment versus control was derived from the ratio between target gene efficiency to the power of target ΔCT and reference gene efficiency to the power of the reference ΔCT.

### 4.4. Immunoblotting

Protein was extracted from the cells using lysis buffer (P0013B, Beyotime Institute of Biotechnology, Shanghai, China) on ice for 1 h and then centrifuged 10,000× *g* for 10 min at 4 °C. The supernatant was collected and a bicinchoninic acid (BCA) protein assay kit (P0012, Beyotime Institute of Biotechnology) was used to measure the protein concentration. Equal amounts of total protein (80 µg) were subjected to sodium dodecyl sulfate/polyacrylamide gel electrophoresis and transferred to polyvinylidene fluoride membranes. The membranes were blocked with 0.5% (*w*/*v*) bovine serum albumin for 0.5 h at 26–28 °C and then incubated overnight at 4 °C with rabbit polyclonal anti-DYT5b primary antibody (1:1000 dilution in blocking buffer, sc-73152, Santa Cruz, CA, USA), rabbit polyclonal anti-DAT primary antibody (1:500 dilution in blocking buffer, sc-14002, Santa Cruz), rabbit polyclonal anti-COMT primary antibody (1:500 dilution in blocking buffer, sc-25844, Santa Cruz Biotechnology, Santa Cruz, CA, USA), goat polyclonal anti-MAO primary antibody (1:500 dilution in blocking buffer, sc-18401, Santa Cruz Biotechnology), goat polyclonal anti-AADC antibody (1:200 dilution in blocking buffer, sc-46909, Santa Cruz Biotechnology), and rabbit polyclonal anti-VMAT2 primary antibody (1:500 dilution in blocking buffer, sc-15314, Santa Cruz Biotechnology), or anti-β-actin primary antibody (1:500 dilution in blocking buffer, YT0099, Immunoway Biotechnology, Plano, TX, USA), respectively. The second day, after washing three times with Tris-buffered saline containing 0.1% (*v*/*v*) Tween-20 at 26–28 °C, the membranes were incubated at 26–28 °C for 1.5 h with alkaline phosphatase goat anti-rabbit secondary antibody or alkaline phosphatase rabbit anti-goat secondary antibody (1:1000 dilution in blocking buffer, AP-1000, AP-5000, both Vector Labs, Burlingame, CA, USA). The membranes were then washed with Tris-buffered saline three times at 26–28 °C and incubated with western blue-stabilized substrate for alkaline phosphatase (S3841, Promega, Madison, WI, USA) for 3 min. The images were recorded with the Tanon-5200 chemiluminescence system (Tanon, Shanghai, China) and analyzed with Quantity One v4.6.2 software (Bio-Rad, Hercules, CA, USA).

### 4.5. ELISA for Dopamine

The dopamine concentrations were measured by a commercially available mouse dopamine ELISA kit (E-EL-0046c, Elabscience Biotechnology Co., Ltd., Wuhan, China). The assay was performed according to the manufacturer’s protocol.

### 4.6. Immunofluorescence

MN9D cells were seeded on a six-well chamber slide for treatment. The cells were then rinsed with PBS and fixed with 4% paraformaldehyde for 15 min at 26–28 °C. The cells were rinsed with PBS, then blocked and permeabilized with 1% normal goat serum and 0.5% TritonX-100 in PBS. The cells were then incubated with the following primary antibodies: mouse monoclonal anti-DYT5b antibody (1:200 dilution in blocking buffer, sc-25269, Santa Cruz Biotechnology), or rabbit polyclonal anti-DAT antibody (1:200 dilution in blocking buffer, sc-14002, Santa Cruz Biotechnology) overnight at 4 °C. The following day after three washes with PBS, the cells were incubated with appropriate secondary antibodies: DyLight 488 anti-rabbit IgG and DyLight 594 anti-mouse IgG (1:200, DI-1088, DI-2594, both Vector Labs, Burlingame, CA, USA) for 40 min at 26–28 °C. The cell nucleus was labeled with 4′,6-diamidino-2-phenylindole (DAPI) (C0065, Solarbio, Beijing, China) for 5 min. After three washes with PBS, the cover slips were sealed with Antifade Mounting Medium (P0128, Beyotime Biotechnology). The results were arranged and analyzed with Image-Pro Plus 6.

### 4.7. Statistical Analysis

The experimental data were analyzed by SPSS18.0 (SPSS, Chicago, IL, USA) and expressed as the mean ± SEM. Factorial design analysis of variance was performed for all data. Dunnett’s multiple comparison tests were used to compare the control and test groups and differences among groups with *p* < 0.05 were considered significant.

## 5. Conclusions

**Figure 8:**
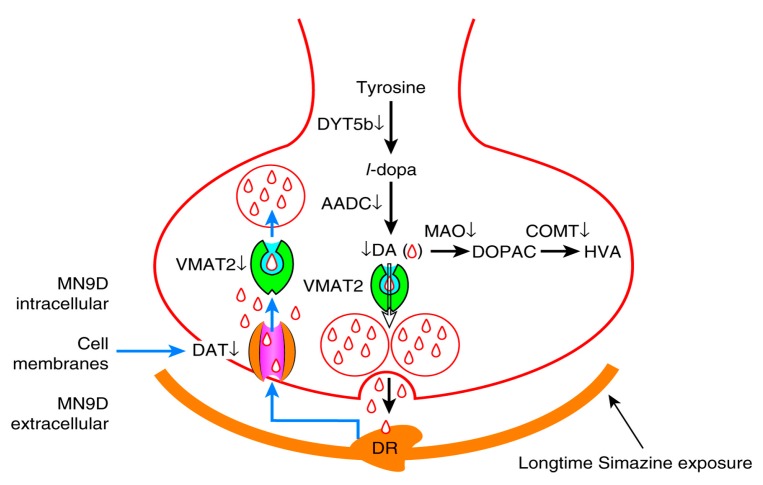
Dopaminergic neuron cell metabolism pathway. DYT5b and AADC participate in the synthesis of dopamine; MAO and COMT degrade a part of redundant dopamine through enzymolysis; DAT and VMAT2 retake and store a part of redundant dopamine in vesicles.

Simazine is neurotoxic and acts on the mammalian dopaminergic metabolism in dopaminergic neurons. Simazine can influence the synthesis, transport and metabolism of dopamine and leads to dysfunction in the homeostasis of the dopaminergic system. Low-dose simazine exposure for 12 and 24 h may increase dopamine levels in MN9D cells. However, the metabolism of dopamine may be damaged in MN9D cells after 48 h due to low-dose simazine exposure. In general, we found that exposure to low-dose simazine, a harmful environmental pollutant, may cause a reduction in dopamine levels after 48 h exposure; thus, we speculate that exposure to low-dose simazine may eventually lead to neurodegenerative diseases (Figure 7 and Figure 8).

## Abbreviations

*l*-DOPA	*l*-3,4-dihydroxyphenylalanine
DYT5b	Tyrosine hydroxylase
AADC	Dihydroxy-Phenyl Acetic Acid
DAT	Dopamine Transporter
VMAT2	Vesicular Monoamine Transporter2
COMT	Catechol-*O*-Methyltransferase
MAO	Monoamine Oxidase
PBS	Phosphate Buffered Solution
SNc	Substantial nigra zona compacta
VTA	Tegmental area
PCR	Reverse transcription-polymerase Chain reaction
CCK-8	Cell Counting Kit-8
ELISA	Enzyme-linked immuno sorbent assay
mRNA	Messenger RNA
cDNA	Completementary DNA
DAPI	4′,6-diamidino-2-phenylindole
PD	Parkinson’s disease
AD	Alzheimer’s disease
